# Tumstatin regulates the angiogenic and inflammatory potential of airway smooth muscle extracellular matrix

**DOI:** 10.1111/jcmm.13232

**Published:** 2017-06-13

**Authors:** Louise Margaret Harkness, Markus Weckmann, Matthias Kopp, Tim Becker, Anthony Wayne Ashton, Janette Kay Burgess

**Affiliations:** ^1^ Respiratory Cell and Molecular Biology Woolcock Institute of Medical Research Sydney NSW Australia; ^2^ Discipline of Pharmacology The University of Sydney Sydney NSW Australia; ^3^ Section for Pediatric Pneumology and Allergology University Medical Center Schleswig‐Holstein Campus Centrum Luebeck Airway Research Centre North (ARCN) Member of the German Centre of Lung Research (DZL) Luebeck Germany; ^4^ Fraunhofer Institute for Marine Biotechnology (Fraunhofer EMB) Luebeck Germany; ^5^ Division of Perinatal Research Kolling Institute of Medical Research Sydney NSW Australia; ^6^ University of Groningen University Medical Center Groningen Department of Pathology and Medical Biology Groningen Research Institute for Asthma and COPD (GRIAC) Groningen The Netherlands

**Keywords:** airway smooth muscle, extracellular matrix, angiogenesis, asthma, collagen IV

## Abstract

The extracellular matrix (ECM) creates the microenvironment of the tissue; an altered ECM in the asthmatic airway may be central in airway inflammation and remodelling. Tumstatin is a collagen IV‐derived matrikine reduced in the asthmatic airway wall that reverses airway inflammation and remodelling in small and large animal models of asthma. This study hypothesized that the mechanisms underlying the broad asthma‐resolving effects of tumstatin were due to autocrine remodelling of the ECM. Neutrophils and endothelial cells were seeded on decellularized ECM of non‐asthmatic (NA) or asthmatic (A) airway smooth muscle (ASM) cells previously exposed to tumstatin in the presence or absence of a broad matrix metalloproteinase inhibitor, Marimastat. Gene expression in NA and A ASM induced by tumstatin was assessed using RT‐PCR arrays. The presence of tumstatin during ECM deposition affected neutrophil and endothelial cell properties on both NA and A ASM‐derived matrices and this was only partly due to MMP activity. Gene expression patterns in response to tumstatin in NA and A ASM cells were different. Tumstatin may foster an anti‐inflammatory and anti‐angiogenic microenvironment by modifying ASM‐derived ECM. Further work is required to examine whether restoring tumstatin levels in the asthmatic airway represents a potential novel therapeutic approach.

## Introduction

Altered ECM composition is a characteristic feature of airway wall remodelling and cardinal to the pathophysiology of asthma 1, 2. The ECM contributes to the microenvironment, which governs cell behaviour, activity, gene transcription and protein expression 2, 3. This microenvironment regulates the behaviour and movement of cells entering airway tissues, including inflammatory cell influxes during inflammation and endothelial cells during angiogenesis. The airway ECM in asthma has been implicated in airway hyper‐responsiveness (AHR), airway thickening, augmented muscle mass, chronic inflammation and increased angiogenesis 4, 5, of which the latter two are the focus of this study.

A distinctive feature of severe asthma is chronic inflammation. Many patients with asthma experience a T‐helper type 2‐mediated response primarily driven by eosinophils 6, 7, which is effectively suppressed by regular use of inhaled corticosteroids (ICS) 8, 9. However, a small subsets of patients (5–10%) are resistant to ICS 10, 11, suffer higher morbidity and mortality, and contribute to the majority of the economic burden associated with asthma 12. Severe asthma features predominately neutrophilic airway inflammation 7, 13, 14. To date, little attention has been paid to the potential of the asthmatic airway ECM microenvironment as an anti‐inflammatory therapeutic tool.

Airway remodelling is a multifactorial and complex process for which the development and persistence may be enhanced by the increased number, size and density of blood vessels. Uninhibited blood vessel expansion promotes tissue dysfunction and uncontrolled cell growth 15 and has been correlated to AHR in asthma 16, 17. Blood vessel growth is regulated by a balance of pro‐ and anti‐angiogenic mediators. Many ECM proteins (including fibronectin, collagen I, collagen IV and thrombospondin) contribute to the maintenance of this balance through their respective pro‐ and anti‐angiogenic properties 4. The dysregulation of these matrix proteins in the asthmatic airway 18, 19 may be one means by which the angiogenic balance is shifted allowing for excessive vascular expansion.

The composition and organization of the ECM microenvironment are tightly regulated in normal tissues 20, 21. Secretion and deposition of ECM proteins are balanced by degradation of matrix filaments by endogenous proteases such as MMPs. In addition, the ECM contributes to the autocrine regulation of these proteases, through direct interactions with cellular surface receptors and *via* matrikines. Matrikines are bioactive ECM fragments which, once released from their parent compound, regulate cellular metabolism to influence ECM deposition and degradation 2, 20. One matrikine of significance in asthma is tumstatin, an anti‐angiogenic fragment of the collagen IV α3 subunit 22, which is a VEGF antagonist 23. Compared to the airways of healthy individuals tumstatin levels are reduced 18‐fold in asthmatic airways 19. Furthermore, administration of tumstatin in large and small animal models of airways disease decreased airway vascularity, reduced airway inflammation and improved AHR 19, 24, revealing a broader functionality of tumstatin in the asthmatic airway.

### Aim of this study

This study aimed to investigate the mechanism of action of tumstatin in airway inflammation and remodelling *via* regulation of the ASM cell‐derived ECM.

## Materials and methods

### Study design

This study aimed to investigate the effect of tumstatin on ASM‐derived ECM‐dependent regulation of airway remodelling and inflammatory response, by examining the behaviour of primary human neutrophils and endothelial cells (human umbilical vein endothelial cells (HUVECs)) reseeded onto the decellularized ECM from non‐asthmatic (NA) or asthmatic (A) ASM cells treated with tumstatin or vehicle control. Real‐time (RT) PCR arrays were used to assess alterations in ASM‐ECM induced by tumstatin. MMP protein expression and activity along with the use of a broad‐spectrum MMP inhibitor were used to assess the role of active MMPs in tumstatin‐induced matrix remodelling.

The key methods and materials used in this study are briefly outlined below. Full details of all methodologies are provided in the online supplement. Information about all the patient derived lung samples used in this study is provided in table S1.

### Tumstatin gene expression by unstimulated primary ASM, lung fibroblasts, lung endothelial cells and airway epithelial cells

Tumstatin gene (COL4A3) expression was assessed in unstimulated NA and A ASM cells, primary lung fibroblasts, primary lung endothelial cells and primary airway epithelial cells from healthy individuals. Exon specific primers for COL4A3 exon 48—exon 49 boundary were used (forward TCATGTCCAGAGGGGACAGT; reverse CCATGTTCATTGGCATCAGA).

### ASM cell Treatment

#### Recombinant human tumstatin

ASM cells were treated with 50 μg/ml recombinant human tumstatin. Tumstatin was produced and purified from *E. coli* colonies as previously described 25. Dialysis buffer from the purification process was used as a vehicle control, which contained equal amounts of endotoxin.

#### Pre‐treatment with broad MMP inhibitor

Marimastat (Santa Cruz Biotechnology Inc., Dallas, TX, USA), a broad MMP inhibitor was reconstituted in DMSO and used in some experiments at 100 μM to pre‐treat cells for 1 hr at 37°C prior to tumstatin treatment. The marimastat was maintained throughout the tumstatin treatment.

### ECM bioactivity assays

#### Chemotaxis of neutrophils seeded onto the decellularized ASM‐ECM

Neutrophils migration was assessed using a μ‐slide (IBIDI, Munich, Germany) coated with the decellularized ECM of tumstatin or vehicle‐treated NA and A ASM cells. Neutrophil migration was monitored relative to a IL‐8 chemoattractant gradient (100 pg/ml in RPMI with 0.5% (w/v) BSA and 2% (w/v) HEPES. A channel coated with 0.001% (w/v) fibronectin acted as a positive control. Neutrophils were imaged every 30 sec. for 1 hr. For individual neutrophils the pathlength, amount of movement towards IL‐8, as well as movement directionality and velocity over the ASM‐ECM were extracted with a Matlab MATLAB algorithm 26, 27. For all experiments 265 neutrophils from five donors and 459 neutrophils from five donors were assessed on the ECM of tumstatin‐ or vehicle‐treated NA ASM cells, respectively. On the tumstatin‐ or vehicle‐treated A ASM‐ECM 107 and 82 neutrophils were assessed, respectively, both from three donors.

#### HUVEC behaviour on the decellularized ASM‐ECM

Human umbilical endothelial cells were seeded onto ECM of tumstatin‐treated NA and A ASM cells, and proliferation and metabolic activities were assessed after 72 hrs using CyQUANT and MTT as per manufacturer's guidelines (Life Technologies, Grand Island, NY, USA and Sigma‐Aldrich, St. Louis, MO, USA, respectively). HUVEC attachment to the ASM‐ECM for 30 min. was assessed with toluidine blue staining 28. HUVECs chemotaxis towards VEGF (10 ng/ml) was performed in a transwell system 29. Migrating cells were stained with toluidine blue and manually counted. These assays were validated by examining HUVEC behaviours on tissue culture surfaces coated with 1 μg/ml fibronectin or gelatin.

## Results

### Tumstatin is not intrinsically expressed by ASM, but is expressed by fibroblasts, endothelial cells and airway epithelial cells

Tumstatin (COL4A3) gene expression was not detectable by untreated NA and A ASM (Fig. S1; *N* = 2 for both groups). Fibroblasts expressed tumstatin at the gene level at baseline (3.03 ± 0.04 expression relative to GAPDH, *N* = 2). Likewise, primary lung endothelial cells and primary airway epithelial cells also expressed tumstatin under non‐stimulatory conditions (2.29 ± 0.13 and 1.66 ± 0.14 expression relative to GAPDH respectively, *N* = 2 and 2).

### Tumstatin suppressed the neutrophil chemotaxis across asthmatic but not non‐asthmatic ASM‐derived matrices

**Figure 1:**
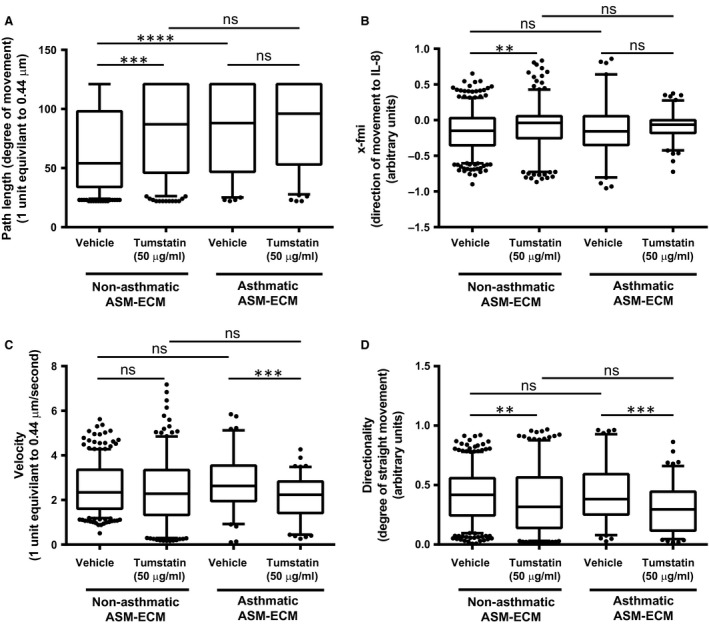
Tumstatin induced an ECM that suppressed neutrophil movement. Migration of neutrophils seeded on the ECM of tumstatin‐ or vehicle‐treated NA (*N* = 4–5) or A ASM (*N* = 3) to IL‐8 was assessed over 1 hr. The path length **(A)**, degree of direct movement to IL‐8 **(**x‐fmi; **B)**, velocity **(C)** and straightness of the neutrophil movement **(**directionality; **D)** were assessed with MATLAB by tracking the movement of individual neutrophils. Data represented as mean and 5–95% percentiles. Groups were compared using a Mann–Whitney test, with **P < 0.01, ***P < 0.001, and ****P < 0.0001. A, asthmatic; ASM, airway smooth muscle; ECM, extracellular matrix; IL, interleukin; NA, non‐asthmatic; x‐fmi, forward movement index in the direction of IL‐8.

Neutrophil movement towards IL‐8 over the ECM from tumstatin‐treated ASM was observed over 1 hr, and recorded on video [Example video files from S1. Non‐asthmatic ASM cell vehicle‐induced matrix; S2. Asthmatic ASM cell vehicle‐induced matrix; S3. Non‐asthmatic ASM cell tumstatin‐induced matrix; S4. Asthmatic ASM cell tumstatin‐induced matrix are provided in the online supplement. The trajectories of individual neutrophils were tracked and characterized (Fig. S2)]. Tumstatin treatment of A ASM cells created an ECM which had no effect on neutrophil movement path length (Fig. 1A; 87.6 ± 3.4 units *versus* the tumstatin vehicle 84.4 ± 4.0 units) nor x‐fmi (amount of forward movement towards IL‐8; Fig. 1B; −0.09 ± 0.02 units *versus* vehicle −0.14 ± 0.04 units) but significantly suppressed both neutrophil velocity (Fig. 1C 2.1 ± 0.09 units *versus* vehicle 2.8 ± 0.14 units, *P* < 0.001) and directionality of neutrophil chemotaxis (ability to move in a straight trajectory; Fig. 1D 0.30 ± 0.019 units *versus* 0.4 ± 0.027 units, *P* < 0.001). The obscure, circular trajectories of neutrophils on the tumstatin‐induced A ASM‐ECM can be seen in Figure S2C, compared with movement along the A ASM‐ECM generated when exposed to the vehicle control (Fig. S2A). Interestingly distance moved by neutrophils over the tumstatin‐induced ECM from NA ASM cells was enhanced (Fig. 1A; 83.0 ± 2.3 units *versus* vehicle 65.0 ± 1.7 units, *P* < 0.001), as was movement towards IL‐8 (Fig. 1B; −0.097 ± 0.020 units *versus* vehicle −0.15 ± 0.013 units, *P* < 0.01). The velocity of movement of neutrophils on the tumstatin‐induced NA ECM was unchanged (Fig. 1C 2.4 ± 0.088 units *versus* vehicle 2.5 ± 0.048 units); however, directionality was decreased (Fig. 1D 0.37 ± 0.016 units *versus* vehicle 0.42 ± 0.0098 units, *P* < 0.01).

The path length travelled by neutrophils on the vehicle‐treated A ASM‐ECM was significantly greater than those on the vehicle‐treated NA ASM‐ECM (Fig. 1A), whilst there were no differences in the x‐fmi, velocity nor directionality of neutrophils on the vehicle‐treated NA or A ASM‐derived matrices (Fig. 1B–D, respectively). These findings suggest a targeted ability of tumstatin to specifically regulate the inflammatory potential of the A ASM‐derived matrix.

### Tumstatin changes the angiogenic potential of the non‐asthmatic and asthmatic ASM‐ECM

**Figure 2:**
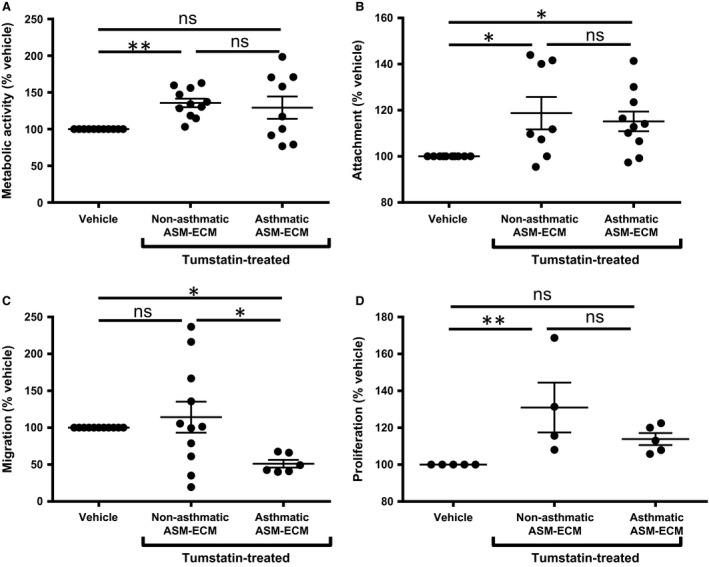
Tumstatin differentially regulates the angiogenic potential of ECM from NA and A ASM cells. HUVECs were seeded on the decellularized ECM of tumstatin‐ or vehicle‐treated NA (*N* = 4–11) or A ASM cells (*N* = 5–10). Metabolic activity **(A)**, attachment **(B)**, chemotaxis of HUVECs through the ECM **(C)** and proliferation **(D)** were assessed. Data represent mean ± S.E.M. and analysed using Wilcoxon and Mann–Whitney tests, with * *P* < 0.05, and **P < 0.01. A, asthmatic; ASM, airway smooth muscle; ECM, extracellular matrix; HUVECs, human umbilical vein endothelial cells; NA, non‐asthmatic.

The ECM deposited by A and NA ASM in the presence of the vehicle control did not induce differences in the behaviour of HUVECs seeded on these matrices in terms of the metabolic activity, attachment nor migration (Fig. S3A–C). The A ASM‐ECM induced greater HUVEC proliferation than the NA ECM (Fig. S3D), reflecting the properties of the A ASM‐ECM previously reported for the induction of ASM cell proliferation 2. Given the similarities in the characteristics of the matrices under basal conditions, we elected to analyse the influence of tumstatin on the ASM‐induced matrices as a percentage of the relevant vehicle control to allow comparisons of responses relative to each cell lines' own baseline, enabling the examination of the overall response pattern between A and NA deposited matrices, whilst taking into account the heterogeneity of the baseline values between the different cell lines (Fig. 2).

The effect that tumstatin‐induced ASM‐ECM remodelling had on the angiogenic potential of the ECM was examined by seeding HUVECs on the tumstatin‐treated ASM‐ECMs. The presence of tumstatin at the time of ECM deposition affected the angiogenic properties of both the NA and A ASM‐derived matrices. Tumstatin induced an ECM from NA ASM cells, but not A ASM cells, that increased HUVEC metabolic activity (NA: 135.7 ± 5.8% vehicle; *N* = 11, *P* < 0.01. A: 129.2 ± 15.3% vehicle, *N* = 9, Fig. 2A). Both the NA and A ASM‐ECM became more adhesive following treatment with tumstatin (NA: 118.8 ± 7.0%vehicle; *N* = 8, *P* < 0.05. A: 115.2 ± 4.3% vehicle; *N* = 10, *P* < 0.05, Fig. 2B), whilst the tumstatin‐treated A ASM‐ECM, but not the NA ASM‐ECM, impaired HUVEC migration (NA: 114.3 ± 21.0% vehicle, *N* = 11, A: 51.1 ± 5.2% vehicle, *N* = 6; *P* < 0.05, Fig. 2C). The tumstatin‐treated NA ASM‐ECM induced greater HUVEC proliferation, whilst there was no change in the proliferative response on the A ASM‐ECM (NA: 130.9 ± 13.5% vehicle, *N* = 4; *P* < 0.01. A: 113.9 ± 3.3% vehicle, *N* = 5, Fig. 2D). There were no differences in the proliferative response between A and NA tumstatin‐induced ASM‐ECM, reflecting the higher basal proliferation on the A ASM‐ECM (Figs 2D and S3D). These data support the hypothesis that restoration of tumstatin in the asthmatic airways may be effective in mitigating aberrant angiogenesis.

### Tumstatin stimulates different genes patterns in non‐asthmatic and asthmatic ASM cells

**Figure 3:**
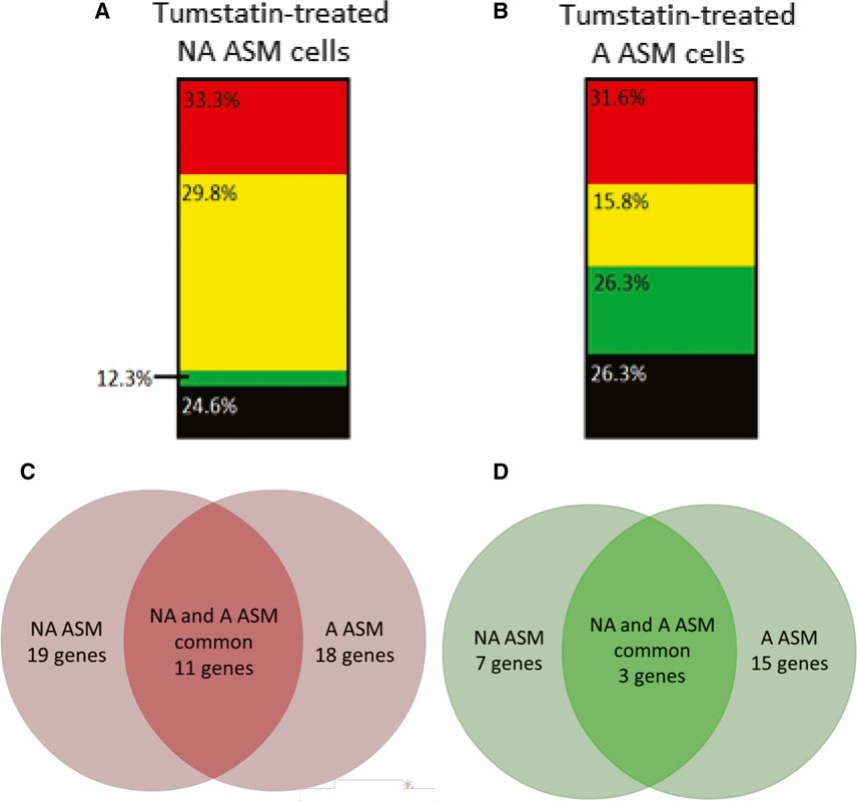
Tumstatin induced different gene expression patterns from NA and A ASM cells. The expression of genes involved in ECM remodelling and angiogenesis in NA (*N* = 3–7) and A ASM (*N* = 3–8) was assessed with RT‐PCR arrays. Genes were determined to have high (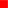 red) or low (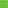 green) levels of expression when showing a ≥ 1.5 fold change (positive or negative respectively) compared to vehicle control. Genes with ≤1.5 fold change were considered to have levels of expression unchanged from baseline (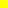 yellow), whilst other genes were not detectable in the ASM cells (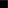 black). The proportion of genes with high, low or baseline levels of expression following treatment of NA **(A)** and A **(B)** ASM cells with tumstatin. Venn diagrams demonstrate the number of common genes with high **(C)** or low **(D)** levels of expression in the NA and A ASM after exposure to tumstatin. A, asthmatic; ASM, airway smooth muscle; ECM, extracellular matrix; NA, non‐asthmatic; RT‐PCR, real‐time PCR.

To explain the shift in the anti‐inflammatory and anti‐remodelling potential of ASM‐derived ECM following tumstatin treatment, we characterized the gene expression patterns of ECM proteins, adhesion molecules and angio‐regulatory molecules in NA and A ASM cells following a 24‐hrs treatment with tumstatin using RT‐PCR arrays. Of the 80 genes investigated 57 were differentially regulated by tumstatin treatment in NA and A ASM cells compared the vehicle control (Figs 3A and B; raw data Table S2). NA ASM cells showed increased expression of 19 (33.3% of all genes) genes, whilst A ASM cells expressed 18 (31.6%) genes higher (Fig. 3A and B, respectively). Almost half the genes regulating inflammation and angiogenesis were up‐regulated (CEACAM‐1, COL4A3, ICAM‐1, IL‐8, ITGA2, PRL, MMP1, MMP10, CXCL10, VCAM‐1) or down‐regulated (KIT, SEMA3F, TIE1) in both NA and A ASM in response to tumstatin (Table S2). These data suggest a common ASM response to tumstatin, although the magnitude of the response was often greater in A ASM (including ICAM‐1, ITGA2, MMP1, MMP10, VCAM‐1, KIT). The more interesting data lay in the differentially expressed genes (Figs 3C, D and Table S2). Genes with greater expression in NA ASM include inflammatory (IFNB1, TNF) and pro‐angiogenic (MDK, PF4, PROK1) cytokines and growth factor receptors (KDR, NCAM) some of which showed >150‐fold difference in expression (Table S2). Conversely, the most highly up‐regulated genes in A ASM include pro‐ (ANGPTL4, TGFA) and anti‐ (LECT1) angiogenic growth factors, and granulocyte chemoattractants (CSF3; Table S2). In addition, in A ASM tumstatin treatment decreased genes not expressed in NA ASM. These genes included potent angiogenic inducers (ANGPT1, ANGPTL3, PDGFB), matrix proteins (THBS3, COL15A1), cell‐cell (SELL) and cell matrix (LYVE1) interactions (Table S2). Overall the reduction of pro‐angiogenic proteins and factors recruiting pro‐resolving macrophages may be why the effects of tumstatin restore homeostasis to asthmatic airways.

**Figure 4:**
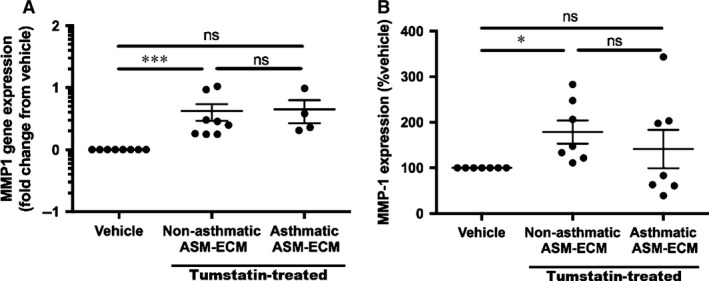
Tumstatin induces MMP‐1 from NA but not A ASM cells. (**A**) MMP‐1 mRNA expression in NA (*N* = 8) and A (*N* = 4) ASM cells by tumstatin compared to the vehicle control. **(B)** MMP‐1 protein was measured in the conditioned media from NA (*N* = 7) or A (*N* = 7) ASM cells treated with tumstatin or the vehicle control. Data are represented as mean ± S.E.M. of fold change from vehicle or % vehicle, and analysed using a Wilcoxon test, and the tumstatin‐treated NA and A ASM were compared with a Mann–Whitney U‐test with *P < 0.05 and ***P < 0.001. A, asthmatic; ASM, airway smooth muscle; MMP, matrix metalloproteinase; NA, non‐asthmatic; RT‐PCR, real‐time PCR.

**Figure 5:**
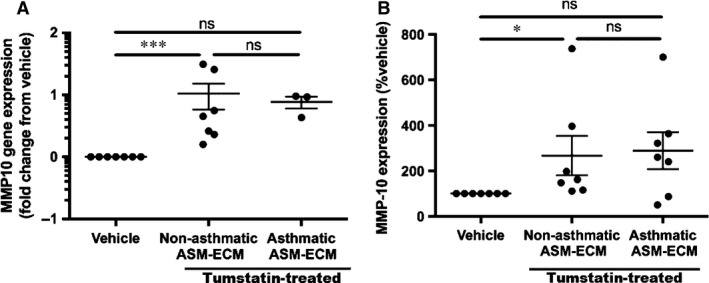
Tumstatin‐induced MMP‐10 from NA but not A ASM cells. **(A)** MMP‐10 mRNA expression by NA (*N* = 7) and A (*N* = 3) ASM cells in response to tumstatin was compared to the vehicle control**. (B)** MMP‐10 protein was measured in the conditioned media from NA (*N* = 7) or A (*N* = 7) ASM cells treated with tumstatin or the vehicle control. Data are represented as mean ± S.E.M. of fold change from vehicle or % vehicle, and analysed using a Wilcoxon test, and the tumstatin‐treated NA and A ASM were compared with a Mann–Whitney U‐test with *P < 0.05 and ***P < 0.001. A, asthmatic; ASM, airway smooth muscle; MMP, matrix metalloproteinase; NA, non‐asthmatic; RT‐PCR, real‐time PCR.

Validation of the top 8 differentially expressed genes (Figs 4, 5, and S4) revealed that only MMP1 (Fig. 4A) and MMP10 (Fig. 5A) were differentially expressed in the cohort of individual ASM samples. Interestingly tumstatin only increased MMP1 and MMP10 levels in NA ASM cells not A ASM cells, at both the gene and protein levels [MMP‐1: Gene: Fig. 4A; 4.2 ± 1.3‐fold change from vehicle, *N* = 8, *P* < 0.01. Protein: Fig. 4B; 178.7 ± 25.5% vehicle, *N* = 7, *P* < 0.05. MMP‐10: Gene: Fig. 5A; 10.5 ± 4.7‐fold change from vehicle, *N* = 7, *P* < 0.05. Protein: Fig. 5B; 266.7 ± 86.7% vehicle, *N* = 7, *P* < 0.05). As the other genes identified in the RT‐PCR arrays were not validated in the larger patient cohort, and they were not characterized further (Fig. S4).

### Tumstatin alters the angiogenic potential of the ASM‐ECM by utilizing active ASM‐derived MMPs

**Figure 6:**
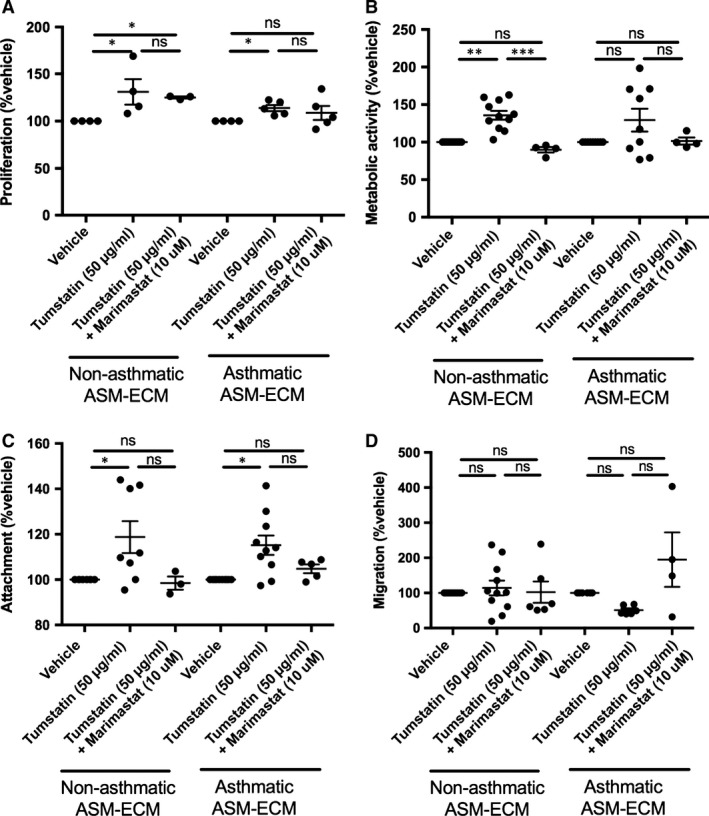
Tumstatin regulated the angiogenic potential of ASM‐derived ECM by utilizing active MMPs. HUVECs seeded on the ECM of NA (*N* = 4–11) or A ASM (*N* = 5–9) cells treated with vehicle, or tumstatin ± Marimastat, were measured for proliferation **(A)**, metabolic activity **(B)**, attachment **(C)**, migration **(D)**. Data represent the mean ± S.E.M. of the change from the vehicle control and analysed using a Kruskal–Wallis test with *P < 0.05, **P < 0.01 and ***P < 0.001. A, asthmatic; ASM, airway smooth muscle; ECM, extracellular matrix; HUVECs, human umbilical vein endothelial cells; NA, non‐asthmatic.

To examine the role of active MMPs in tumstatin‐driven matrix remodelling on the angiogenic potential of ASM‐ECM, we pre‐incubated NA and A ASM cells with the broad‐spectrum MMP inhibitor Marimastat (100 μM) prior to tumstatin and examined the angiogenic potential of the ECM produced. Proliferation of HUVECs on ECM from tumstatin‐treated NA ASM cells was unaffected by Marimastat (Fig. 6A; tumstatin 130.9 ± 13.5%, *N* = 4, Marimastat pre‐treatment 125.0 ± 1.1% appropriate vehicle, *N* = 3), whilst Marimastat reversed the increased metabolic activity of HUVECs on the tumstatin‐treated NA ASM‐ECM (Fig. 6B; tumstatin 89.8 ± 3.6%, *N* = 11, Marimastat pre‐treatment 137.5 ± 5.8% appropriate vehicle, *N* = 4, *P* < 0.001). The tumstatin induced increase in adhesiveness of the NA ASM‐ECM was reversed in the presence of the MMP inhibitor (Fig. 6C NA: tumstatin 118.8 ± 7.0%, *N* = 8, Marimastat pre‐treatment 98.5 ± 2.9% appropriate vehicle, *N* = 3, A: tumstatin 104.3 ± 1.9% *N* = 10, Marimastat pre‐treatment 115.2 ± 4.3% appropriate vehicle, *N* = 5). On the matrices from the tumstatin‐treated A ASM the proliferation and adhesion were reversed by pre‐treatment with Marimastat (Proliferation: Fig. 6A; Marimastat pre‐treatment 108.7 ± 7.4% appropriate vehicle, *N* = 5. Adhesion: Fig. 6C; Marimastat pre‐treatment 104.8 ± 1.9% appropriate vehicle, *N* = 5). These data indicate that the mechanism by which tumstatin drives ASM‐derived ECM remodelling is only partly through the use of active MMPs.

## Discussion

Tumstatin is a matrikine not detectable in asthmatic airway tissues but which is present in airway tissues of people without asthma. When introduced to animal models of asthma tumstatin resolves features of airway remodelling and inflammation and improves lung function 19, 24. The current study characterized the role of tumstatin in the maintenance of airway tissue homeostasis, through regulation of the ASM‐derived ECM, and the potential contribution of the absence of tumstatin in asthmatic airways to the pathogenesis. Tumstatin induces an ASM‐derived‐ECM, which provides a microenvironment with anti‐inflammatory and anti‐angiogenic characteristics.

Tumstatin gene expression was detected in primary lung fibroblasts, endothelial cells and airway epithelial cells, but not in primary ASM cells. This reflects the distribution of tumstatin protein, we have previously reported in human lung tissue 19. Given the absence of tumstatin production by ASM cells, this study investigated the exogenous effects of this matrikine that is potentially released from the non‐asthmatic ECM to regulate tissue homeostasis.

Through the examination of neutrophil movement across matrices from ASM cells exposed to tumstatin, the role of tumstatin in regulating the inflammatory properties of the airway wall ECM microenvironment was investigated. When reintroduced to A ASM cells, tumstatin induced an ECM which disrupted neutrophil motility, slowing the rate and decreasing the ability of neutrophils to move purposefully towards a chemoattractant. This, to our knowledge, is the first illustration of the ECM providing directional cues for neutrophil migration in asthmatic lung. Similar disruptions in neutrophil movement were seen on the tumstatin‐induced NA ASM‐ECM, in addition to a decrease in the purposeful movement, or directionality of the neutrophils despite increased pathlength. Neutrophils use cell‐surface receptors and integrins to transmigrate through the endothelial cell layer and identify, attach to, and migrate through the localized tissue 30. The influence of the tissue ECM on neutrophil infiltration remains poorly understood. It is known that neutrophil proliferation is enhanced by fibrinogen 31 and survival increased by fibronectin and laminin 32. Laminin enhances protease release from neutrophils and neutrophil chemotaxis 33 potentiating inflammatory cell movement. The current study findings suggest tumstatin has the potential to create an anti‐inflammatory microenvironment, which inhibits neutrophils in the airway tissue.

The bioactive properties of tumstatin‐induced ASM‐ECM were assessed further, by examining the angio‐regulatory properties of the ASM‐ECM following exposure to tumstatin. Tumstatin induced an ECM from NA and A ASM cells capable of differential regulation of endothelial cell behaviour. The tumstatin‐stimulated NA ASM cell microenvironment increased the proliferation, metabolic activity and attachment of endothelial cells, whilst the ECM from tumstatin‐treated A ASM cells enhanced endothelial cell attachment but impaired movement. Blood vessel expansion, specifically the process of vessel sprouting, requires the formation of a highly specific and motile differentiated tip cell and the proliferation of stalk cells 1, 34. *In vivo*, the tip cells must begin migration before the proliferation of the stalk cells is purposeful. The consequence of the increased attachment and decreased chemotaxis of endothelial cells on the tumstatin‐treated ASM‐matrices would be the failure of sprout formation. The subsequent limitation of new tubule formation combined with the ASM‐ECM would hence hamper the angiogenic process. Thus, an anti‐angiogenic matrix was deposited from both NA and A ASM cells in the presence of tumstatin.

Changes to the bioactive properties of the ECM can result from matrix remodelling. Proteases, once released and activated, act on the ECM, altering the structure, composition and thus biological function. Two proteases, MMP‐1 and ‐10, were expressed highly in NA ASM cells following tumstatin treatment. The use of the broad‐spectrum MMP inhibitor in this study which inhibited the tumstatin‐induced NA ASM‐ECM enhanced metabolic activity and proliferation, suggested these proteases were partly responsible for the anti‐angiogenic properties that tumstatin induced in the NA ASM‐ECM. These data are in concert with earlier observations of MMPs regulating inflammatory properties of the ECM. MMP9‐overexpressing transgenic mice exhibited altered leucocyte extravasation and reduced lymphocyte accumulation in the airway walls 35. In addition, in our study tumstatin altered the expression of a number of genes, from both NA and A ASM cells, involved in MMP activation (such as SERPIN B5), although the gene expression patterns were different, and experiments were not carried out to validate gene array findings.

ASM gene expression in the presence or absence of tumstatin was assessed broadly with RT‐PCR arrays, exploring tumstatin's effect on genes involved in ECM remodelling and angiogenesis. There were a number of common genes induced or reduced in the NA and A ASM cells following tumstatin treatment, suggested a core response in healthy and diseased ASM to this matrikine. One significant example is the large increase in expression of collagen IV α3 (COL4A3) induced by tumstatin in both NA and A ASM cells. This gene encodes for the parent protein of tumstatin, suggesting reintroduction of tumstatin into the airway tissue initiates a positive feedback loop increasing the release of the active matrikine. These strong positive feedback systems, in addition to the therapeutic anti‐inflammatory and anti‐remodelling effects of tumstatin *in vitro* and *in vivo,* hint at the vital role of tumstatin for airway homeostasis.

### Implications of this study

This study provides new knowledge enabling a better understanding of the beneficial anti‐remodelling and anti‐inflammatory effects of tumstatin when administered to the lungs. This study elucidates a mechanism through which tumstatin modulates the ECM microenvironment as a broad influential regulator of multiple cell types and cell behaviours. Tumstatin drives specific characteristics of the ASM‐ECM that may control the expansion of the airway vasculature and inflammatory cell influx in the airway tissue. This study expands previous reports of tumstatin as a potential asthma therapeutic.

## Conflict of interest

The authors have no competing interests to report related to the production of these data or the preparation of this manuscript.

## Supporting information


**Figure S1** Primary lung endothelial cells, airway fibroblasts and airway epithelial cells express tumstatin.

**Figure S2** Tumstatin induces A but not NA ASM cells to deposit an ECM which disrupts the movement of neutrophils.

**Figure S3** HUVECS respond similarly to NA and A ASM‐ECM deposited in the presence of tumstatin vehicle.

**Figure S4** Validation of genes expressed by NA and A ASM cells in response to tumstatin.

**Video S1** Nonasthmatic airway smooth muscle cell vehicle‐induced matrix.

**Video S2** Asthmatic airway smooth muscle cell vehicle‐induced matrix.

**Video S3** Nonasthmatic airway smooth muscle cell tumstatin‐induced matrix.

**Video S4** Asthmatic airway smooth muscle cell tumstatin‐induced matrix.

**Appendix S1** Materials and methods.
**Table S1** Information of samples used in this study.
**Table S2** Tumstatin differentially regulates gene expression patterns in NA and A ASM cells.
