# Supplementary figures and images for: Tumstatin regulates the angiogenic and inflammatory potential of airway smooth muscle extracellular matrix
Source: J Cell Mol Med. 2017 Jun 13;21(12):3288–97. doi: 10.1111/jcmm.13232 (PMC5706579; doi:10.1111/jcmm.13232)

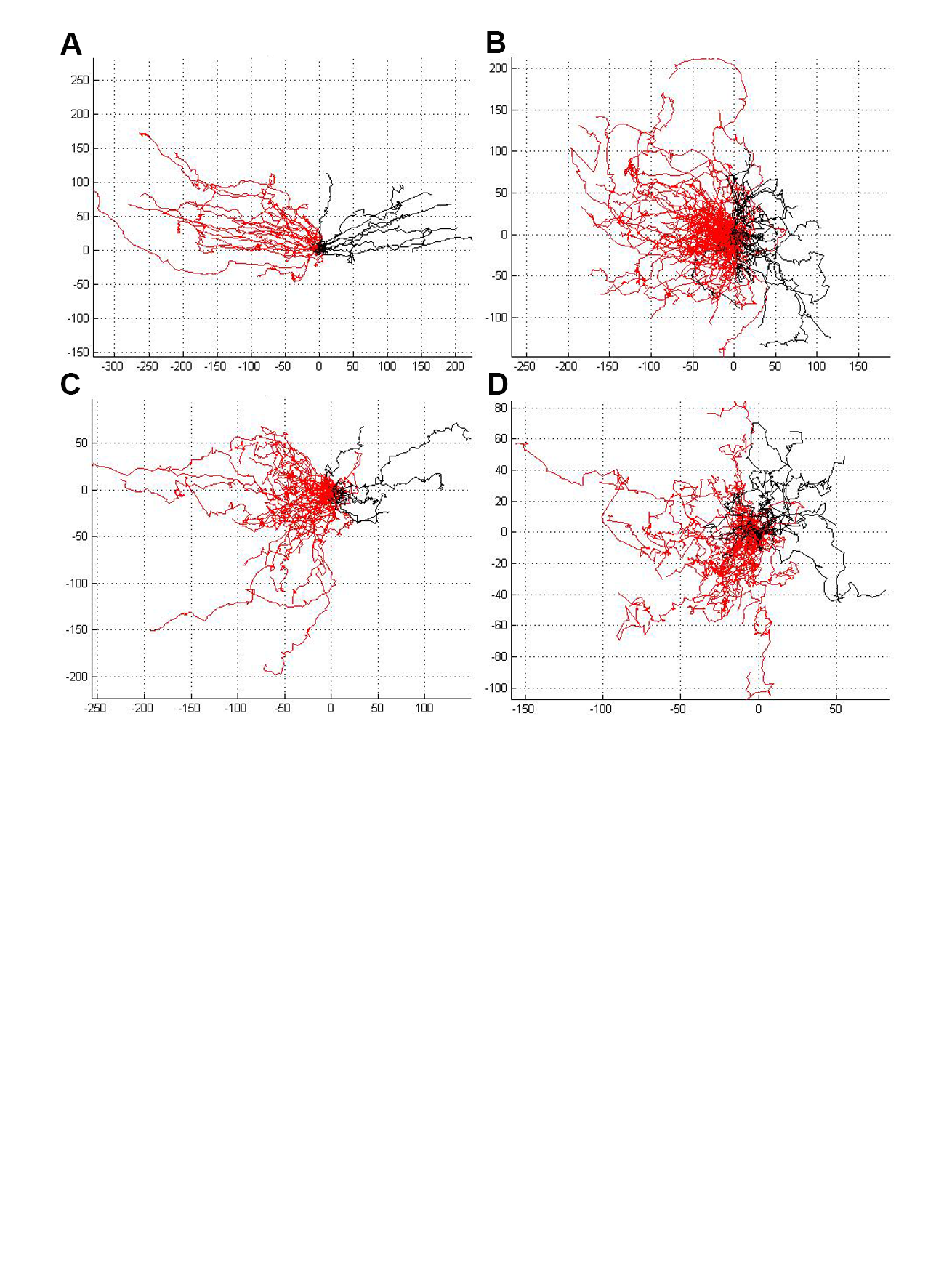

Supplement: Supplementary file 2 — Figure S2 Tumstatin induces A but not NA ASM cells to deposit an ECM which disrupts the movement of neutrophils. [file JCMM-21-3288-s002.tif]
