# Supplementary material for: Tumstatin regulates the angiogenic and inflammatory potential of airway smooth muscle extracellular matrix
Source: J Cell Mol Med. 2017 Jun 13;21(12):3288–97. doi: 10.1111/jcmm.13232 (PMC5706579; doi:10.1111/jcmm.13232)
Supplement: Supplementary file 9 — Appendix S1 Materials and methods. Table S1 Information of samples used in this study. Table S2 Tumstatin differentially regulates gene expression patterns in NA and A ASM cells. [file JCMM-21-3288-s009.docx]

# TUMSTATIN REGULATES THE ANGIOGENIC AND INFLAMMATORY POTENTIAL OF AIRWAY SMOOTH MUSCLE EXTRACELLULAR MATRIX

**Online Supplement**

Harkness, Louise Margaret. ^1,2^, Weckmann, Markus^3^, Kopp, Matthias^3^, Becker, Tim^4^, Ashton, Anthony Wayne^5^, Burgess, Janette Kay^1,2,6^*

^1^ Respiratory Cell and Molecular Biology, Woolcock Institute of Medical Research. Sydney, NSW, Australia.

^2^ Discipline of Pharmacology, The University of Sydney. Sydney, NSW, Australia.

^3^ Section for Pediatric Pneumology and Allergology, University Medical Center Schleswig-Holstein, Campus Centrum Luebeck, Airway Research Centre North (ARCN), Member of the German Centre of Lung Research (DZL), Germany.

^4^ Fraunhofer Institute for Marine Biotechnology (Fraunhofer EMB), Luebeck, Germany

^5^ Division of Perinatal Research, Kolling Institute of Medical Research, Sydney, NSW, Australia.

^6^ University of Groningen, University Medical Center Groningen, Department of Pathology and Medical Biology, GRIAC Research Institute, Groningen, the Netherlands.

* Assoc Prof Janette Burgess

University Medical Center Groningen

Department of Pathology and Medical Biology

Medical Biology Section

Hanzeplein 1

[IPC EA11, Rm z2.7]

9713 GZ Groningen

The Netherlands

phone:+31-50-3618043 (secr) / +31-50-3610985

fax: +31-50-3619911

email: j.k.burgess@umcg.nl

# Materials and Methods

## Ethics

For lung tissues, written informed consent was provided by individuals (or the next of kin) undergoing research bronchoscopy, scheduled lung resection, or lung transplantation. ‘Macroscopically normal’ was isolated by the hospital pathologist. The Human Research Ethics Committee (HREC) of the South West Sydney Area Health Service, Royal Prince Alfred Hospital, and the University of Sydney provided approval for this study (Approval numbers AU/1/76B9015, X14-0045, 5201300355, 11507, and 10139). For HUVEC isolation, informed consent was obtained from pregnant women prior to delivery by caesarean section. Ethics approval was obtained from the Northern Sydney Local Health District HREC (1004-145M –17/05/2010). Neutrophil isolation from the blood of non-asthmatic individuals was done so with approval from the HREC of the University of Luebeck Medical School (AZ11-044). Research was carried out according to the World Medical Association Declaration of Helsinki.

## Study population and primary cell isolation and culture

Bronchial tissue was obtained from a total of 19 individuals who answered yes to the question, “has a doctor ever told you that you have asthma?” and 24 healthy volunteers or ‘macroscopically normal’ tissue from non-asthmatic carcinoma patients. Full characteristics of the participants are provided in table 1A. ASM cells were microdissected from the lung tissue as previously described[1], fibroblasts were grown from diced human lung tissue as described [2] and primary airway epithelial cells were grown from bronchial brushing obtained during bronchoscopy or from the epithelial layer removed from the airways by macrodissection.[3]. ASM cells and fibroblasts negative for the presence of Mycoplasma were used between passages 2 and 6 or 2 and 5 respectively.

Isolation of Lung endothelial cells

Primary lung endothelial cells were isolated from healthy lung tissue using a protocol adapted from [4]. Lung tissue was minced and incubated in digestion buffer (250 U/ml type 2 collagenase, 10 mg/ml BSA, 1.25% (w/v) pancreatin and 1 U/ml dispase in DMEM) for 30 min at 37°C. Liberated endothelial cells were isolated using anti-CD31 dynabeads and cultured on gelatine-coated cultureware in 15% (v/v) FCS, 20 U/ml heparin, 10 µg/ml ECGS, 1 μg/ml hydrocortisone, and 100 U/ml antibiotic/antimitotic in MCDB131. All materials from Life Technologies, (CA, USA) unless stated otherwise.

### Airway smooth muscle cell culture

ASM cells from non-asthmatic (NA) or asthmatic (A) individuals were seeded into tissue culture surfaces at 1x10^4^ cells/cm^2^ in DMEM (Life Technologies, CA, USA) supplemented with 10% FBS (DKSH, VIC, AUS), 100 U penicillin, 100 µg/mL streptomycin (Ab), and 25mM HEPES (Life Technologies, CA, USA) for 72h. Growth arrest was induced by switching to DMEM supplemented with 0.1% BSA (Sigma Aldrich, St Louis, MO, USA), Ab and 25mM HEPES (arrest media) for 48h. Following a 24h treatment (outlined below) ASM cells were either collected in lysis buffer for mRNA extraction or decellularised ASM-ECM generated by incubating cultures with 0.016mM NH_4_OH at 37^o^C for 30-60 min with 3 washes in 1x PBS before and after.

### Neutrophil isolation

Neutrophils were isolated from human blood collected in syringes containing lithium-heparin coated beads (Sarstedt, Nümbrecht, Germany) and laid on top of 2.5 volumes of lymphocyte separation medium (Histopaque-1077; Sigma, Taufkirchen, Germany). Erythrocytes and neutrophils were pelleted with a cooled centrifugation at 1,200 rpm for 30min with brake removed. Erythrocytes were lysed by resuspending the pellet in 45mL of cold Ampuwa water (Fresenius Kabi, Bad Homburg, Germany) for 40s before 5mL of chilled 10x PBS was added and cell suspension was centrifuged at 1,100 rpm for 10min with brake on at 8^o^C. Neutrophils were checked for viability using manual cell counts in the presence of trypan blue (used when ≥95% viable) and activation by the exclusion of propidium iodide solution (Sigma, Taufkirchen, Germany) and CD62L; Beckman Coulter, Krefeld, Germany).

### Human Umbilical Vein Endothelial Cells

Primary HUVECs were isolated from healthy mothers as previously described and grown in M199 with 5U/mL Penicillin/Streptomycin, 2mM L-Glutamine (Sigma-Aldrich, MO, USA), 20% heat inactivated new born calf serum (NBCS), 5% heat inactivated human serum (Sigma-Aldrich MO, USA), 45µg/mL BBE (Lonza, Basel, Switzerland), 7.5µg/mL ECGs (Sigma-Aldrich MO, USA), and 10U/mL heparin (Sigma-Aldrich MO, USA) (referred to as HUVEC culture media). All materials from Life Technologies, (CA, USA) unless stated otherwise. After passage 0 HUVECs from 3 to 10 umbilical cords were pooled. HUVECs were used at passages 1-5 for experiments.

**Tumstatin gene expression by untreated ASM, fibroblasts, and endothelial cells**

## Tumstatin gene (COL4A3) expression was assessed in untreated primary lung cells. ASM cells from NA and A ASM cells were examined after 96h and 168h incubations in growth media, while primary lung fibroblasts, lung endothelial and epithelial cells from healthy individuals, were examined after a 24h incubation in basal medium. mRNA was extracted from these cells using a NucleoSpin RNA kit as per manufacturer’s instructions (Macherey-Nagel, Düren, Germany), and reverse transcription was performed using random hexameric primers and SuperScript III (Invitrogen, USA). Relative expression of COL4A3 (tumstatin gene) was quantified using exon specific primers for COL4A3 exon 48 – exon 49 boundary (forward TCATGTCCAGAGGGGACAGT; reverse CCATGTTCATTGGCATCAGA). Gene expression was normalised to human GAPDH using log_2_∆Ct method[5].

## ASM cell Treatment

### Recombinant human tumstatin

ASM cells were treated with 50µg/mL recombinant human tumstatin. Tumstatin was produced and purified from *E. coli* colonies as previously described [6]. Dialysis buffer from the purification process was used as a vehicle control which contains equal amounts of endotoxin.

### Pre-treated with broad MMP inhibitor

Marimastat (Santa Cruz Biotechnology Inc., CA, USA), a broad MMP inhibitor was reconstituted in DMSO and used in some experiments at 100µM to pre-treat cells for 1h at 37^o^C prior to tumstatin treatment. Marimastat was maintained at the same concentration throughout the tumstatin treatment.

## ASM Experimental Output:

### Real-time PCR

ASM mRNA was extracted using an ISOLATE RNA Mini kit (Bioline, NSW, AUS) as per the manufacturer’s instructions and quantified with a Nanodrop 1000 Spectrophotometer (NanoDrop Technologies, Inc. Wilmington, DE, USA). Reverse transcription was performed on an Eppendorf PCR Thermocycle (Eppendorf, Hamburg, Germany) using M-MLV and corresponding reagents (Life Technologies, Carlsbad, CA, USA) according to the manufacturer’s instructions.

Equal amounts of cDNA were pooled from NA (N=3) and A (N=3) ASM cells to create a single NA and A ASM sample for assessment by RT-PCR TaqMan® Array for Human ECM & Adhesion Molecules (#4414133; Applied Biosystems, VIC, AUS) and as per the manufacturer’s instructions. A similar approach was used to assess NA (N=7) and A (N=8) ASM samples using the TaqMan Human Angiogenesis ® Array (#4414071).

Validation of array findings for MMP-1 and MMP-10 were conducted on NA (N=8 and 8) and A (N=4 and 3 respectively) ASM cells with primers Hs00899658_m1 and Hs00233987_m1, respectively (Life Technologies, Carlsbad, CA, USA) using as per the manufacturer’s guidelines SensiFast MasterMix (Bioline, NSW, AUS) and StepOne Plus detection system (Thermo Fisher, CA, USA). Relative gene expression was calculated by using the ∆∆Ct method[5] and StepOne software (Thermo Fisher, CA, USA), normalised to human 18S expression.

### Protease protein expression

Total MMP-1 and -10 secreted from NA or A ASM cells, treated with or without tumstatin, were measured using Luminex Performance Assay kits (R&D Systems, Minneapolis, MN, USA) according to the manufacturer’s instructions, and quantified using a Luminex analyser (Luminex 200 System; Luminex, QLD, AUS).

## ECM bioactivity assays

### Chemotaxis of neutrophils seeded onto the decellularised ASM-ECM

After resting at 37^o^C for 2h, neutrophils (3x10^6^ cells/mL) were added to the centre channel of a µ-slide (IBIDI, Munich, Germany) previously coated with the decellularised ECM of ASM cells as outlined above, and allowed to adhere for 30min at 37^o^C. The µ-slide was brought to room temperature before 100pg/mL IL-8 (BD Bioscience, Munich, Germany) in RPMI (Biochrom AG, Berlin, Germany) supplemented with 0.5% BSA (Sigma, Taufkirchen, Germany), 2% HEPES (Thermo Fisher, CA, USA) was added to the left reservoir and the vehicle control (RPMI 0.5% BSA, 2% HEPES) to the right reservoir. A channel coated with 0.001% fibronectin (Sigma, Taufkirchen, Germany) acted as a positive control. An EVOS Fluorescence microscope (Thermo Fisher Scientific, Darmstadt, Germany) imaged neutrophil chemotaxis every 30s for a period of 1h. Using Matlab (MathWorks, MA, USA) for each individual neutrophil the path length, amount of movement toward IL-8, as well as movement directionality and velocity over the ASM-ECM was extracted with a Matlab algorithm [7].

### HUVEC behaviour on the decellularised ASM-ECM

HUVECs were quiesced for 36h in supplement-free HUVEC culture media (without additional BBE, ECGs, or heparin) and then seeded onto ECM derived from tumstatin-treated NA and A ASM cells in fresh supplement-free HUVEC culture media. HUVEC proliferation and metabolic activity were assessed 72h after seeding (1x10^5^ cells/mL) using CyQUANT and MTT as per manufacturers’ guidelines (Life Technologies, CA, USA and Sigma, MO, USA, respectively). For attachment HUVEC (1x10^5^ cells/mL) were plated onto ASM-ECM for 30min at 37^o^C and quantified with toluidine blue staining as previously described [8] using a Spectromax M2 and Soft Max pro software (version 4.8 Molecular Devices, Sunnyvale, CA, USA). HUVECs chemotaxis toward VEGF (10ng/mL) was performed in a transwell system (8μm pore size) [9]. Migrating cells were stained with toluidine blue, after fixation, and mounted onto slides in 70% glycerol/1x PBS and quantified by manual counts in 5 fields of view (FOV) per membrane randomly selected using an Olympus BX60 light microscope and Olympus DP71 camera (Olympus, Tokyo, Japan). These assays were validated by examining HUVEC behaviours on tissue culture surfaces coated with 1μg/mL fibronectin or gelatin

References

1. **Johnson PRA, Roth M, Tamm M*, et al.*** Airway smooth muscle cell proliferation is increased in asthma. *Am J Respir Crit Care Med*. 2001; 164: 474-7.

2. **Krimmer DI, Burgess JK, Wooi TK*, et al.*** Matrix proteins from smoke-exposed fibroblasts are pro-proliferative. *Am J Respir Cell Mol Biol*. 2012; 46: 34-9.

3. **Ge Q, Moir LM, Black JL*, et al.*** TGFbeta1 induces IL-6 and inhibits IL-8 release in human bronchial epithelial cells: the role of Smad2/3. *J Cell Physiol*. 2010; 225: 846-54.

4. **Sobczak M, Dargatz J, Chrzanowska-Wodnicka M.** Isolation and culture of pulmonary endothelial cells from neonatal mice. *J Vis Exp*. 2010.

5. **Livak KJ, Schmittgen TD.** Analysis of relative gene expression data using real-time quantitative PCR and the 2(-Delta Delta C(T)) Method. *Methods*. 2001; 25: 402-8.

6. **Faiz A, Tjin G, Harkness L*, et al.*** The expression and activity of cathepsins D, H and K in asthmatic airways. *PLoS One*. 2013; 8: e57245.

7. **Rapoport DH, Becker T, Madany Mamlouk A*, et al.*** A Novel Validation Algorithm Allows for Automated Cell Tracking and the Extraction of Biologically Meaningful Parameters. *PLoS ONE*. 2011; 6: e27315.

8. **Moir LM, Black JL, Krymskaya VP.** TSC2 modulates cell adhesion and migration via integrin-alpha1beta1. *Am J Physiol Lung Cell Mol Physiol*. 2012; 303: L703-10.

9. **Ashton AW, Ware JA.** Thromboxane A2 receptor signaling inhibits vascular endothelial growth factor-induced endothelial cell differentiation and migration. *Circ Res*. 2004; 95: 372-9.

**Table S1. Information of samples used in this study.** The demographical information of samples were collected from consenting patients is outlined in the table below.

| **No.** | **Diagnosis** | **Gender** | **Age (years)** | **Smoking history** | **Sample** | **FEV_1_** | **FVC** | **Experiment** |
| --- | --- | --- | --- | --- | --- | --- | --- | --- |
| 1 | Asthma | F | 42 | N | Bronchoscopy | - | - | 11, 12 |
| 2 | Asthma | M | 25 | - | Bronchoscopy | - | - | 1, 5, 16 |
| 3 | Asthma | F | 18 | N | Bronchoscopy | 3.21L | 3.49L | 4, 11, 12, 15 |
| 4 | Asthma | M | 21 | N | Bronchoscopy | - | - | 3, 4, 8, 14, 15 |
| 5 | Asthma | M | 20 | N | Bronchoscopy | 5.09L | 6.24L | 3, 6, 8, 9, 10, 11, 12, 14 |
| 6 | Asthma | M | 23 | N | Bronchoscopy | 2.80L | 4.47L | 6, 8, 9, 10, 11, 12 |
| 7 | Asthma | M | 22 | N | Bronchoscopy | 3.80L | 5.16L | 2, 3, 4, 5, 13, 14, 15, 16 |
| 8 | Asthma | M | 21 | N | Bronchoscopy | 4.45L | 5.00L | 3, 6, 7, 11, 12, 14 |
| 9 | Asthma | M | 45 | N | Bronchoscopy | 4.00L | 5.36L | 1, 7, 8, 9, 10, 11, 12 |
| 10 | Asthma | F | 56 | N | Bronchoscopy | 2.04L | 2.50L | 3, 4, 5, 14, 15, 16 |
| 11 | Asthma | F | 50 | N | Bronchoscopy | 1.87L | 2.32L | 2, 4, 5, 13, 15, 16 |
| 12 | Asthma | M | 27 | N | Bronchoscopy | 3.75L | 5.90L | 2, 13 |
| 13 | Asthma | M | 22 | - | Bronchoscopy | - | - | 2, 3, 4, 5, 13, 14, 15, 16 |
| 14 | Asthma | M | 21 | N | Bronchoscopy | 3.12L | 4.82L | 3, 4, 7, 14, 15 |
| 15 | Asthma | M | 39 | - | Bronchoscopy | - | - | 3, 4, 7, 8, 14, 15 |
| 16 | Asthma | F | 19 | - | Bronchoscopy | 91% | - | 7, 8 |
| 17 | Asthma | M | 23 | - | Bronchoscopy | 82% | 81% | 3, 4, 9, 11, 12, 14, 15 |
| 18 | Asthma | F | 27 | - | Bronchoscopy | 2.66L | 3.83L | 4, 5, 7, 8, 15, 16 |
| 19 | Asthma | M | 24 | - | Bronchoscopy | 73% | 78% | 2, 13 |
| 20 | Non-asthmatic | M | 22 | N | Bronchoscopy | - | - | 8 |
| 21 | Non-asthmatic | M | 31 | N | Resection | - | - | 8, 11, 12 |
| 22 | Normal healthy control | M | 20 | - | Bronchoscopy | - | - | 3, 5, 14, 16 |
| 23 | Normal healthy control | F | 27 | N | Bronchoscopy | - | - | 5, 8, 10, 11, 12, 16 |
| 24 | Normal healthy control | F | 21 | N | Bronchoscopy | - | - | 5, 6, 8, 9, 10, 11, 12, 16 |
| 25 | Normal healthy control | M | 69 | - | Bronchoscopy | - | - | 3, 6, 8, 9, 10, 11, 12, 14 |
| 26 | Normal healthy control | M | 27 | N | Bronchoscopy | - | - | 7, 9 |
| 27 | Normal healthy control | F | 22 | Ex | Bronchoscopy | 91% | 86% | 8, 9, 10 |
| 28 | Normal healthy control | F | 22 | - | Bronchoscopy | - | - | 3, 4, 14, 15 |
| 29 | Donor- trauma | - | - | - | - | - | - | 3, 5, 14, 16 |
| 30 | Donor - trauma | M | 48 | N | Transplant | - | - | 1, 3, 5, 6, 7, 8, 9, 10, 11, 12, 14 |
| 31 | Ca | F | 50 | - | Resection | - | - | 5, 16 |
| 32 | Ca | M | 66 | Y | Resection | 88% | 92% | 1 |
| 33 | NSSCa | M | 71 | N | Resection | 93% | 89% | 2, 3, 4, 13, 14, 15 |
| 34 | NSCCa | M | 68 | Ex | Resection | 82% | 78% | 1, 3, 5, 13, 16 |
| 35 | NSSCa | M | 71 | Ex | Resection | 79% | 86% | 1 |
| 36 | NSCCa | F | 58 | N | Resection | 96% | 91% | 3, 4, 5, 15, 16 |
| 37 | NSCCa + malignant neoplasm | M | 59 | Ex | Resection | 76% | 73% | 3, 4, 7, 14, 15 |
| 38 | NSCCa + malignant neoplasm | F | 73 | Ex | Resection | 100% | 113% | 4, 7, 8, 9, 10, 15 |
| 39 | NSCCa + malignant neoplasm | F | 56 | Ex | Resection | 66% | 71% | 4, 7, 8, 9, 10, 11, 12, 15 |
| 40 | Squamous cell Ca + adenocarcinoma  + malignant neoplasm | M | 75 | Ex | Bronchoscopy | 85% | 79% | 5, 7, 9, 10, 11, 12, 16 |
| 41 | Metastatic melanoma | F | 38 | - | Resection | - | - | 3, 14 |
| 42 | Adenocarcinoma | F | 59 | N | Resection | 111% | 106% | 3, 4, 5, 13, 14, 15, 16 |
| 43 | No diagnosis | M | 58 | N | Transplant | - | - | 3, 4, 5, 14, 15, 16 |

**Experiments**: Neutrophil migration on the decellularized ECM of NA and A ASM cells **(1)**. HUVEC on ASM decellularized ECM: Proliferation **(2)**, metabolic activity **(3)**, attachment **(4)**, and migration **(5)**. RT-PCR arrays: Human ECM proteins and adhesion molecules **(6)**, Human Angiogenesis array **(7)**.Validation RT-PCR assays **(8)**. Follow-up of MMP-1 **(9)** and MMP-10 **(10)** gene transcription and MMP-1 **(11)** and MMP-10 **(12)** protein expression. HUVEC behavioural assays on the tumstatin-induced NA or A ASM-ECM deposited in the presence of a broad MMP inhibitor: Proliferation **(13)**, metabolic activity **(14)**, attachment **(15)**, and migration **(16)**.

**Abbreviations**: A: asthmatic, ASM: airway smooth muscle, Ca: carcinoma, Ex: ex-smoker, FEV_1_: Forced Expiratory Volume in the first second (% predicted values or litres), FVC: Forced Vital Capacity (% predicted values or litres), N: no, NA: non-asthmatic, NSSCa: non-small cell Ca, RT-PCR: real-time PCR.

**Table S2. Tumstatin differentially regulates gene expression patterns in NA and A ASM cells.** Modulation of gene expression in quiescent NA and A ASM cells was analysed *in vitro* following a 24h stimulation with tumstatin (50μg/mL). ASM cells were lysed, mRNA extracted and pooled samples were run on Human ECM, Adhesion Molecule and Angiogenesis RT-PCR arrays (Thermo Fisher, CA, USA). Gene expression was determined by the ∆∆Ct method [5] compared to the vehicle control.

| **Gene** | **Tumstatin-treated NA ASM** | **Tumstatin-treated A ASM** |  | **Gene** | **Tumstatin-treated NA ASM** | **Tumstatin-treated A ASM** |  | **Gene** | **Tumstatin-treated NA ASM** | **Tumstatin-treated A ASM** |
| --- | --- | --- | --- | --- | --- | --- | --- | --- | --- | --- |
| **ANG** | * | * |  | **ICAM1** | 4.3 | 13.8 |  | **PF4** | 1.9 | - |
| **ANGPT1** | - | 0.3 |  | **IFNB1** | 3.4 | * |  | **PLG** | * | * |
| **ANGPT2** | * | * |  | **IL8** | 58.5 | 3.9 |  | **PRL** | 20.0 | 1.9 |
| **ANGPT4** | - | 1.6 |  | **ITGA2** | 1.9 | 6.7 |  | **PROK1** | 19.7 | 0.1 |
| **ANGPTL3** | * | 0.2 |  | **ITGB4** | - | * |  | **PROX1** | 1.8 | 1.5 |
| **CHGA** | * | * |  | **KDR** | 2.2 | - |  | **PTN** | - | - |
| **CTNND2** | 0.6 | * |  | **KIT** | 0.4 | 0.2 |  | **SEMA3F** | 0.3 | 0.3 |
| **CEACAM1** | 6.9 | 1.9 |  | **LAMB3** | - | 6.5 |  | **SELE** | * | * |
| **CDH1** | - | 0.3 |  | **LECT1** | - | 1.6 |  | **SELL** | - | 0.3 |
| **CDH5** | - | - |  | **LYVE1** | - | 0.4 |  | **SELP** | * | * |
| **COL4A3** | 2.8*10^5^ | 1.4*10^5^ |  | **MDK** | 1.6 | - |  | **SERPINB5** | * | 0.2 |
| **COL15A1** | - | 0.6 |  | **MMP1** | 3.5 | 12.0 |  | **SERPINF1** | 1.8 | - |
| **CTNNB1** | - | 6.5 |  | **MMP10** | 3.5 | 8.4 |  | **TGFA** | 0.4 | 1.9 |
| **CSF3** | 0.3 | 7.9 |  | **MMP8** | * | * |  | **THBS3** | - | 0.5 |
| **CXCL10** | 109.9 | 3.2 |  | **MMP9** | * | * |  | **TIE1** | 0.1 | 0.4 |
| **CXCL12** | - | * |  | **MMP13** | * | * |  | **TNF** | 1.5 | 0.2 |
| **FGA** | * | * |  | **NCAM1** | 2.0 | 0.3 |  | **TNNI1** | 0.1 | 6.0 |
| **FGF1** | - | - |  | **PDGFB** | * | 0.3 |  | **VCAM1** | 8.7 | 24.5 |
| **FST** | - | - |  | **PDGFRA** | - | - |  | **VTN** | * | * |

Increased and decreased determined by a fold change in gene expression which is 1.5 higher or lower than vehicle, * not detectable, - no change.
**Abbreviations**: A: asthmatic, ASM: airway smooth muscle, , ECM: extracellular matrix, NA: non-asthmatic, RT-PCR: real-time PCR.

**Supplementary figures**

**Figure S1. Primary lung endothelial cells, airway fibroblasts and airway epithelial cells express tumstatin.** The expression of tumstatin gene (COL4A3) was assessed by RT-PCR in primary lung endothelial cells, primary lung fibroblasts, primary airway epithelial cells and primary ASM cells. Data are presented as mean±SD relative to expression of the housekeeping gene GAPDH. Statistical analysis was not performed due to low sample size (n=2 for each cell type).

**Figure S2. Tumstatin induces A but not NA ASM cells to deposit an ECM which disrupts the movement of neutrophils.** The trajectories for each individual neutrophil moving across the vehicle-treated A **(A)** or NA **(B)** ASM-ECM were tracked and compared to the movement of neutrophils over the ECM from A **(C)** or NA **(D)** ASM cells treated tumstatin. A Matlab (MathWorks, MA, USA) algorithm was used to track individual neutrophils. Each neutrophil was realigned to (0,0), movement toward IL-8 (on the left) was recorded in red and movement away from the chemoattractant was marked in black.
**Abbreviations**: A: Asthmatic, ASM: airway smooth muscle, ECM: extracellular matrix, NA: non-asthmatic.

**Figure S3. HUVECS respond similarly to NA and A ASM-ECM deposited in the presence of tumstatin vehicle.** NA (N=4-11) and A ASM cells (N=5-10) were treated with tumstatin vehicle for 24h before the deposited ECM was decellularised. HUVECs were seeded onto the ASM-derived ECMs and allowed to migrate toward VEGF. Proliferation (A), metabolic activity (B), attachment (C), and chemotaxis of HUVECs through the ECM (D) where assessed. Data represent mean±SD. Groups were compared using an unpaired t-test Friedman test with *P<0.05.
**Abbreviations:** A: asthmatic, ASM: airway smooth muscle, ECM: extracellular matrix, NA: non-asthmatic, VEGF . vascular endothelial growth factor.

**Figure S4. Validation of genes expressed by NA and A ASM cells in response to tumstatin.** mRNA expression by NA and A ASM cells following a 24h treatment with tumstatin was compared to the vehicle control using quantitative RT-PCR as validation of the ECM, Adhesion Molecule, and Angiogenesis RT-PCR arrays. ANGPT1 **(A)**, LAMB3 **(B)**, PROK1 **(C)**, CXCL10 **(D)**, CTNNB1 **(E)**, VCAM1 **(F)** expression levels were assessed and data presented as mean±SEM fold from vehicle control. Groups were compared with a Wilcoxon test with * P<0.05.
**Abbreviations**: A: asthmatic, ASM: airway smooth muscle, ANGPT1: angiopoietin 1, CTNNB1: catenin β1, ECM: extracellular matrix, LAMB3: laminin β3 subunit, NA: non-asthmatic, PROK1: prokineticin 1, RT-PCR: real-time PCR.

**Supplementary video files**

Images captured every 30s over a 60min period.

Neutrophil migration towards IL-8 (left of screen) along:

1. Nonasthmatic airway smooth muscle cell vehicle-induced matrix

2. Asthmatic airway smooth muscle cell vehicle-induced matrix

3. Nonasthmatic airway smooth muscle cell tumstatin-induced matrix

4. Asthmatic airway smooth muscle cell tumstatin-induced matrix
